# Prevention of bile duct injury using indocyanine green fluorescence in laparoscopic liver cyst fenestration for giant liver cyst: a case report

**DOI:** 10.1093/jscr/rjac479

**Published:** 2022-10-19

**Authors:** Tomonari Shimagaki, Shinji Itoh, Katsuya Toshida, Takahiro Tomiyama, Akinari Morinaga, Yukiko Kosai, Takahiro Tomino, Takeshi Kurihara, Yoshihiro Nagao, Kazutoyo Morita, Noboru Harada, Tomoharu Yoshizumi

**Affiliations:** Department of Surgery and Science, Graduate School of Medical Sciences, Kyushu University, Fukuoka, Japan; Department of Surgery and Science, Graduate School of Medical Sciences, Kyushu University, Fukuoka, Japan; Department of Surgery and Science, Graduate School of Medical Sciences, Kyushu University, Fukuoka, Japan; Department of Surgery and Science, Graduate School of Medical Sciences, Kyushu University, Fukuoka, Japan; Department of Surgery and Science, Graduate School of Medical Sciences, Kyushu University, Fukuoka, Japan; Department of Surgery and Science, Graduate School of Medical Sciences, Kyushu University, Fukuoka, Japan; Department of Surgery and Science, Graduate School of Medical Sciences, Kyushu University, Fukuoka, Japan; Department of Surgery and Science, Graduate School of Medical Sciences, Kyushu University, Fukuoka, Japan; Department of Surgery and Science, Graduate School of Medical Sciences, Kyushu University, Fukuoka, Japan; Department of Surgery and Science, Graduate School of Medical Sciences, Kyushu University, Fukuoka, Japan; Department of Surgery and Science, Graduate School of Medical Sciences, Kyushu University, Fukuoka, Japan; Department of Surgery and Science, Graduate School of Medical Sciences, Kyushu University, Fukuoka, Japan

## Abstract

The case is a 78-year-old female. A giant liver cyst was pointed out by abdominal echo from 7 years ago, but because the size of the cyst tended to increase, it was decided to operate taking into account the risk of the cyst rupturing. Laparoscopic surgery was started, and the cyst contents did not fluorescent when observed by the indocyanine green (ICG) fluorescence method. Laparoscopic liver cyst fenestration was performed using the ICG fluorescence method, paying attention to the damage to the bile duct excluded by the cyst. The opened cyst was filled with the greater omentum. In this report, we describe that the ICG fluorescence method can evaluate the presence or absence of bile leakage from the hepatic dissection and the running of the bile duct on the inner wall of the cyst, and is considered to contribute to safer laparoscopic liver cyst fenestration.

## INTRODUCTION

Nonparasitic liver cysts are congenital or acquired and are considered to be caused by an aberrant bile duct that has lost its communication with the normal biliary tree, which ultimately results in an isolated fluid-collected cavity in the liver [1]. In most cases, liver cysts remain asymptomatic and do not require treatment [1]. However, treatment is required when the cyst increases in diameter and/or the patient experiences symptoms such as pain and abdominal distension. Surgical treatments include fenestration procedures, liver resection and liver transplantation [2]. One of the surgical advantages that can be attributed to laparoscopic fenestration (LF) is the sufficient fenestration that prevents the recurrence of the cyst. However, excessive resection of the cyst wall may induce severe complications, such as bile leakage from the cut edge of the liver cyst [3]. This is because the fine bile ducts are not visible through the cyst wall in the majority of cases, and it is difficult to recognize all of them during fenestration.

Indocyanine green (ICG) clearance test is a well-established and one of the most reliable preoperative liver function tests [4]. It is also known that ICG administered into blood is excreted in the bile [5]. This property has been used in a study to observe ICG excreted into the biliary tract for avoiding bile duct injury in a laparoscopic cholecystectomy [5]. In this report, we describe a case of successful LF in the patient with symptomatic giant liver cysts using ICG to avoid bile duct injury, along with a review of literature.

## CASE REPORT

**Figure 1:**
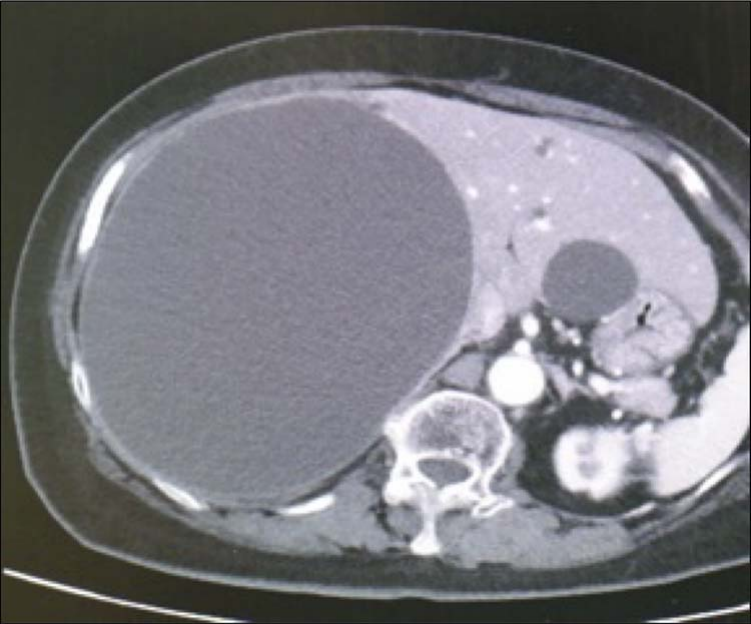
Preoperative computed-tomography imaging demonstrating giant liver cyst (axial section).

A 78-year-old female patient was followed up by a family doctor who pointed out a giant liver cyst (maximum diameter 11.5 cm) by abdominal echo 7 years ago. Her liver cyst was gradually increasing (maximum diameter 17 cm; Fig. 1), and she was at risk of cyst rupture, so she was referred to our hospital for surgical purposes. The laboratory data revealed no specific findings.

**Figure 2:**
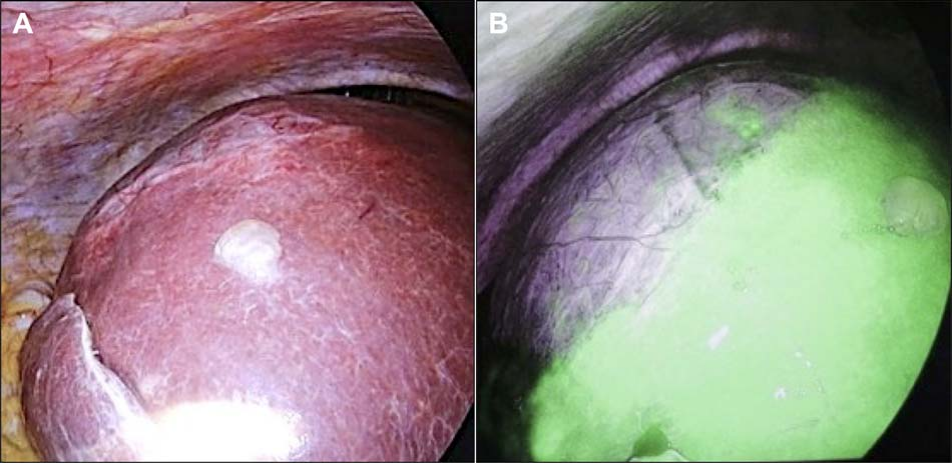
Intraoperative findings. (**A**) A giant cyst was confirmed in the abdominal cavity. (**B**) At ~1 h after ICG injection, the liver parenchyma showed strong fluorescence signals.

**Figure 3:**
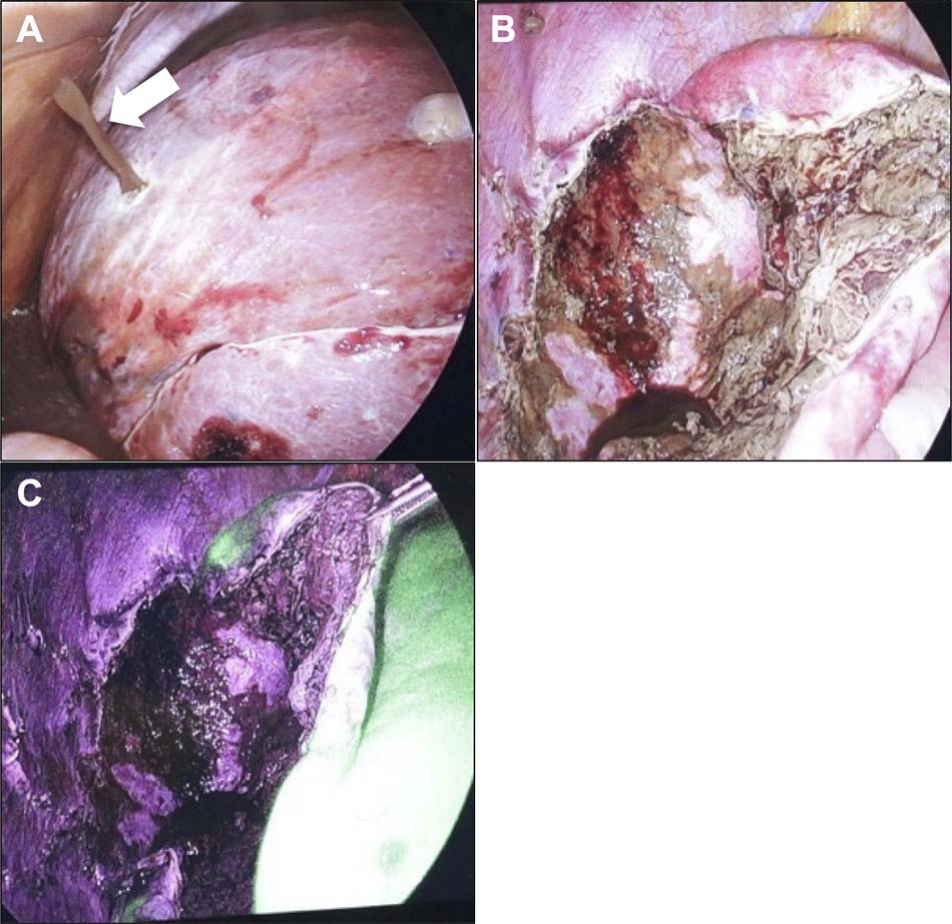
Intraoperative findings. (**A**) When puncturing a cyst, an outflow of brown cyst fluid was observed (arrow). Confirmation at the bottom of the cyst after fenestration procedure. Images obtained under normal light (**B**) and near-infrared light (**C**). There was no evidence of bile leakage upon macroscopic observation.

Initially, 2.5 mg of ICG was administered intravenously just after induction of anesthesia. A 12-mm trocar was inserted at the umbilicus with a laparoscope, followed by the placement of three ports for the operation. We used the VISERA ELITE video system (Olympus Corporation, Japan) with a designated rigid scope (IR Laparoscope®, Stryker, Kalamazoo, Michigan, USA), which could provide fusion fluorescence imaging consisting of normal and near-infrared light images. Cysts were observed on and inside the liver (Fig. 2A and B). ICG fluorescence was detected in the liver parenchyma and the biliary tract, but not in the cyst wall (Fig. 2B). The cyst contents were punctured and drained outside the body. The cystic fluid did not exhibit ICG fluorescence (Fig. 3A). ICG fluorescence imaging clearly distinguished the cysts from the liver parenchyma, and we could resect only the cyst wall as wide as possible under the guidance of white light and fluorescence imaging using LigaSure (Medtronic, Dublin, Ireland; Fig. 3B). After completing the deroofing procedure, we confirmed that there was no ICG fluorescence (implying bile leakage) based on macroscopic observation against the bottom of the cyst under near-infrared light view (Fig. 3C). Intracystic bile ducts and bile leaks in the cysts were not detected during surgery. Finally, the opened cyst was filled with the greater omentum and then the abdomen was closed. The patient was discharged 5 days after surgery without any complication. Histopathological examination revealed that the cyst wall consisted of fibrous tissue without any signs of malignancy.

## DISCUSSION

Liver cysts are relatively common with a prevalence of 2–7% among the population and are typically discovered incidentally [6]. The pathogenesis of liver cysts is uncertain; however, as they are commonly observed in women, a correlation with estrogen levels has been suggested, and symptomatic liver cysts are more common in females [7].

Surgical options for treating symptomatic liver cysts include fenestration, hepatectomy and liver transplantation [2]. Other treatment options include needle aspiration, which is associated with a high recurrence rate, and sclerotherapy such as ethanol injection [8]. Since the first report of LF for liver cysts in 1991 [9], this procedure has been used as the standard method, although it has the risk of postoperative bile leakage [3].

In addition to various liver function tests, ICG tests are generally performed to evaluate the safety of hepatectomy, and these tests are also adopted, for example, as criteria for assessing the degree of liver damage. The ICG test was introduced and popularized by Makuuchi *et al*. in the 1980s, especially in patients with hepatocellular carcinoma [10]. In patients with normal liver function, 95% of ICG is excreted into the bile duct after absorption by hepatocytes within 15 min [11]. In patients with impaired liver function, the excretion rate of ICG decreases and the timing of ICG when it can be observed in the bile would be delayed. The use of ICG in hepatobiliary surgery has become common, and its use to identify the biliary tree in hepatobiliary surgery has been reported [12].

Usually, for visualizing a tumor, ICG is administered 24–48 h before the surgery [13]. On the other hand, for fluorescent cholangiography, ICG is intravenously administered 0.5–24 h before the surgery [5]. In previous report, intravenous administration of ICG 1 h before the surgery as sufficient to successfully visualize the small bile duct in the cyst wall, and it also allowed us to avoid accidental bile duct injury [14]. We followed this procedure based on a previous report that the maximum concentration of ICG in bile juice was observed within 2 h after intravenous administration [15].

## CONCLUSION

LF using intravenously administrated ICG that can visualize the intrahepatic bile duct located in the cyst wall is an effective method to reduce the risk of accidental bile duct injury during LF. Further study is needed to optimize the timing of ICG administration to confirm whether ICG fluorescence can help prevent and manage bile duct injury during LF. ICG fluorescence–guided LF has the potential to be used as a standard surgical procedure for treating symptomatic liver cysts.
